# Are Volatile Organic Compounds Accurate Markers in the Assessment of Colorectal Cancer and Inflammatory Bowel Diseases? A Review

**DOI:** 10.3390/cancers13102361

**Published:** 2021-05-13

**Authors:** Filippo Vernia, Marco Valvano, Stefano Fabiani, Gianpiero Stefanelli, Salvatore Longo, Angelo Viscido, Giovanni Latella

**Affiliations:** Gastroenterology Unit, Department of Life, Health and Environmental Sciences, University of L’Aquila, Piazza S. Tommasi, Coppito, 67100 L’Aquila, Italy; filippo.vernia1@gmail.com (F.V.); valvano.marco@libero.it (M.V.); sfabiani92@gmail.com (S.F.); giastefanelli@gmail.com (G.S.); salvator.longo@gmail.com (S.L.); angelo.viscido@univaq.it (A.V.)

**Keywords:** volatile organic compounds, VOCs, colorectal cancer, inflammatory bowel diseases, IBD, ulcerative colitis, Crohn’s disease, microbiome

## Abstract

**Simple Summary:**

Early diagnosis is crucial for reducing colorectal cancer-related mortality in both the general population and inflammatory bowel disease. Volatile organic compound (VOC) analysis is a promising alternative to the gold standard procedure, endoscopy, for early detection and surveillance of colorectal diseases. This review aimed to provide a general overview of the most recent evidence in this area on VOC testing in breath, stool, and urine samples.

**Abstract:**

Colorectal cancer (CRC) is one of the leading causes of cancer-related death in the Western world. Early detection decreases incidence and mortality. Screening programs based on fecal occult blood testing help identify patients requiring endoscopic examination, but accuracy is far from optimal. Among the alternative strategies, volatile organic compounds (VOCs) represent novel potentially useful biomarkers of colorectal cancer. They also represent a promising tool for the screening of both intestinal inflammation and related CRC. The review is focused on the diagnostic potential of VOCs in sporadic CRC and in inflammatory bowel diseases (IBD), which increase the risk of CRC, analyzing future clinical applications. Despite limitations related to inadequate strength of evidence, differing analytical platforms identify different VOCs, and this unconventional approach for diagnosing colorectal cancer is promising. Some VOC profiles, besides identifying inflammation, seem disease-specific in inflammatory bowel diseases. Thus, breath, urine, and fecal VOCs provide a new and promising clinical approach to differential diagnosis, evaluation of the inflammatory status, and possibly the assessment of treatment efficacy in IBD. Conversely, specific VOC patterns correlating inflammatory bowel disease and cancer risk are still lacking, and studies focused on this issue are strongly encouraged. No prospective studies have assessed the risk of CRC development by using VOCs in samples collected before the onset of disease, both in the general population and in patients with IBD.

## 1. Introduction

Colorectal carcinoma (CRC), with an estimated incidence of 1.4 million patients/year, is one of the most common malignancies worldwide [1]. Prevention and early diagnosis reduce mortality, both in sporadic CRC and inflammatory bowel disease (IBD)-related CRC with a five-year survival approaching 90% [1]. Thus, identification and treatment of preneoplastic lesions in the general population and dysplasia in IBD are crucial. As bowel symptoms and fecal tests are non-specific [2], endoscopic screening represents the gold standard for early diagnosis of CRC [3]. However, being invasive, endoscopy is not well accepted by a large proportion of patients, reducing the effectiveness of screening programs [4] and the efficacy of the therapeutic options.

The same limitations apply to other conditions requiring endoscopic monitoring such as IBD, in which indirect markers of activity and dysplasia are still inadequate or lacking [5,6]. In these patients, burdened by a higher risk of CRC compared to the general population, endoscopy is particularly important [7], but non-invasive, reliable, simple, and possibly inexpensive alternatives are needed. To date, however, none of the current options fulfills these requirements in CRC and IBD.

The microbiome is central in both CRC and IBD, and changes in bacterial strain composition affect the pattern of fermentation products, organic anions, and volatile organic compounds (VOCs) [8]. Thus, metabolomics, reflecting the cellular metabolism, provides an alternative approach for diagnosis and the monitoring of disease course, as different conditions are associated with specific metabolomic profiles [9]. Indeed, in IBD, the decreased Firmicutes to Bacteroidetes ratio is linked to colonic inflammation and influences the production of compounds including short-chain fatty acids (SCFA) and VOCs [10,11]. Similarly, several VOCs correlate with bacterial taxa in both inactive and active Crohn’s disease (CD) [12]. The same applies to CRC, in which several bacterial strains seem to exert a pro-carcinogenic effect [13]. Therefore, as changes in bacterial strain composition are different in CRC and IBD, theoretically, the VOC signatures between CRC and IDB should also be different. However, no studies have directly evaluated and compared the VOC signatures in patients with CRC and IBD.

**Figure 1:**
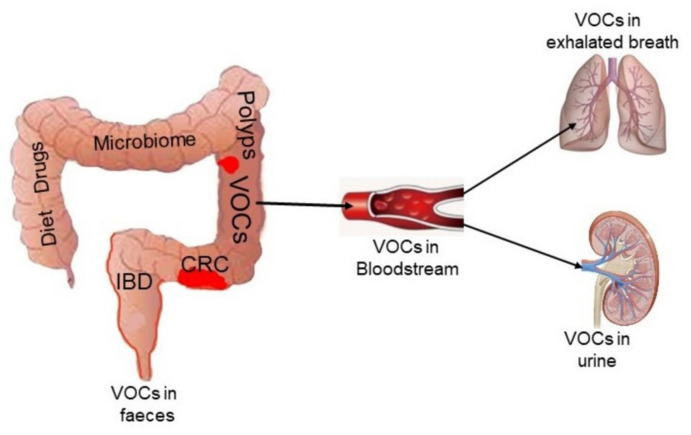
Volatile organic compounds (VOCs) are produced during physiological and pathological processes including colonic inflammation and neoplasia. VOCs can also originate from exogenous sources such as food and drugs and microbiome metabolism. Once produced, VOCs are released into the gut and detectable in the intraluminal content or feces, or released into the bloodstream and then reach the pulmonary alveoli or renal tubules where they are excreted and therefore measurable in the exhaled breath and urine, respectively. IBD = inflammatory bowel disease; CRC = colorectal cancer.

VOCs are usually defined as volatile carbon-containing organic molecules that can be directly detected in feces or diffuse into the bloodstream and be subsequently eliminated through the lungs (in the exhaled breath) or the kidneys (in the urine) [14] (Figure 1). According to their origin, VOCs may be divided into exogenously or endogenously derived compounds. Exogenous VOCs originate from the environment including diet and smoking. Endogenous VOCs are end-products of human or microbial metabolism. However, this definition is not universally accepted, as some authors also include chemicals that are gaseous at room temperature [15], or not particularly volatile compounds such as amino acids [16].

The origin of individual VOCs is still a matter of debate, but largely depends on the interaction between diet and microbiota. The relationship is complex and bidirectional in normal subjects as gluten-free diet [17], ketogenic diets [18], low FODMAP diet [19] as well as alcohol intake [20] influence both the microbiome and VOC production. However, it is not known to what extent the various components of the diet favor the production of VOCs more than non-volatile organic anions such as SCFA and vice versa.

The VOC pattern results from complex interactions that differ in physiologic and pathologic conditions. The metabolic processes are indeed affected by mucosal changes, necrosis included, and the ensuing alteration of the intestinal microbiome. It has thus been anticipated that VOC analysis may prove helpful in different clinical settings including diagnosis and monitoring of disease course. Analysis of VOCs, being non-invasive, inexpensive, and well-tolerated, may represent an alternative to endoscopy [21]. A growing interest is thus focusing on conditions in which repeated endoscopy is required such as colorectal cancer screening, follow-up of benign preneoplastic lesions in the general population, and monitoring the disease course or identifying patients at high risk for dysplasia in IBD.

However, examining the potential clinical role of VOC analysis is complex as studies have been carried out using different analytical techniques. VOC analysis uses two main techniques: gas chromatography-mass spectrometry (GC-MS) and electronic noses. The first provides a chemical analysis of specific compounds, while the second needs to be “trained” to recognize specific breath patterns using machine learning [22,23].

An eNose consists of different arrays of electronic chemical sensors combined with an appropriate pattern-recognition system [24]. VOCs react on the surface of the sensors, inducing a change in conductivity that is detected by transducers and converted into electrical signals [24]. Several distinct eNose technologies have been developed including metal-oxide sensors (Aeonose, PEN3) [24], electrochemical and optical sensors (WOLF) [15], and conducting-polymer sensors (Cyranose 320) [25]. The different arrays and technologies, however, may result in a wide variety of chemical class coverage and accuracy. The identification and analysis of molecules by GC-MS are accurate but complex and time-consuming. Thus, eNoses have recently been preferred in experimental studies.

This narrative review aims to provide a general overview of published evidence on VOCs analysis in CRC and IBD and critically analyze their clinical potential as well as future perspectives.

## 2. Materials and Methods

A systematic electronic search of the literature (without language or data publication restriction) was performed using Medline/PubMed, Embase, Scopus, and Web of Science up to February 2021. The search included a combination of medical subject headings (MeSH) and keywords as follows:

(“volatile organic compounds” [MeSH Terms] OR “volatile organic metabolites” [All Fields] OR “VOC” [All Fields] OR “VOM” [All Fields]) AND ((“colorectal neoplasms” [MeSH Terms] OR “colorectal neoplasia” [All Fields] OR “colorectal cancer” [All Fields] OR “digestive system neoplasms” [MeSH Terms] OR “colonic neoplasms” [MeSH Terms]) OR (“inflammatory bowel diseases” [MeSH Terms] OR “colitis, ulcerative” [MeSH Terms] OR “Crohn disease” [MeSH Terms] OR “IBD” [All Fields] OR (“UC” [All Fields] OR “CD” [All Fields]).

Three authors (FV, MV, SF) independently reviewed the abstract and full text to assess the eligibility. Conflicts were resolved by consensus, referring to the original articles. The reference section of each relevant publication was also screened for other publications. Prospective and retrospective cohort studies, case-control studies, and analytical cross-sectional studies were considered. In vitro and animal studies were excluded.

**Figure 2:**
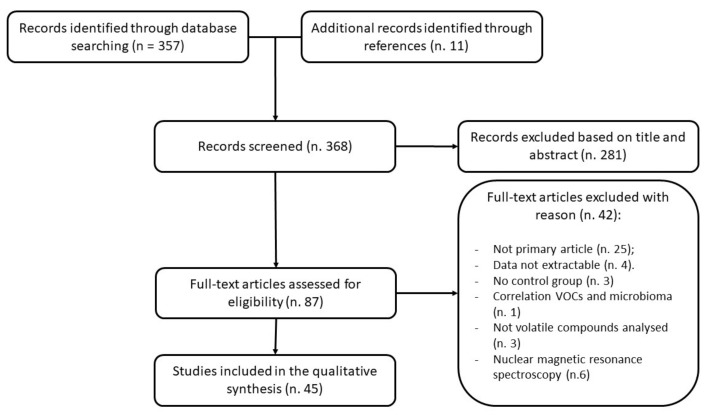
The PRISMA diagram reports the reasons for the exclusion of the articles.

Out of 368 articles, 281 records were excluded based on their titles and abstracts, 42 for specific reasons, and 45 full text included (Figure 2).

The specific VOCs identified in the included studies are reported in Table 1 for breath exhaled compounds, and in the caption of each table for fecal and urinary VOCs.

## 3. Results

### 3.1. Colorectal Cancer

#### 3.1.1. Breath Exhaled VOCs

The detection of CRC represents one of the main applications of technologies analyzing breath-exhaled VOCs. Studies identified several different potentially useful compounds, but different techniques led to differing results (Table 2).

Altomare et al. [33] reported that a profile of 15 VOCs identified with GC-MS could discriminate between CRC patients and healthy controls with an accuracy exceeding 80%. The same group recently reported that the use of new advanced breath samplers, capturing only the alveolar breath fraction and excluding environmental contaminants, improved the overall accuracy of 14 exhaled VOCs with a sensitivity and a specificity of 90 and 93%, respectively (Table 1) [26].

These results are in line with those obtained by a group using a GC-MS, which reported sensitivity of 85%, specificity of 94%, and accuracy of 91% [30].

Conversely, an Italian study using a commercial e-nose (PEN3™ eNose—Airsense Analytics GmbH, Schwerin, Germany), was unable to discriminate between CRC patients and healthy controls despite the higher detection of methane, methane derivatives, organic and aromatic compounds, in CRC patients [29]. More recently, however, good accuracy was reported using a different eNose (Aeonose™—The eNose Company, Zutphen, the Netherlands) [23], which showed an AUC of 0.84, with a sensitivity of 95% and specificity of 64%, respectively. Interestingly, the authors [23] also reported promising results in the detection of advanced adenomas with an AUC of 0.73, 79% sensitivity, and 59% specificity. The eNose was, however, unable to differentiate between CRC and advanced adenomas.

The different results obtained using different eNoses might derive from lower sensitivity to CRC-associated VOCs of PEN3™ eNose sensors than that of Aeonose™ sensors. There are, however, no studies directly comparing the accuracy in detecting CRC-associated breathprints of the two devices.

It has also been reported that breath analysis of VOCs can discriminate patients with adenomas from healthy controls [23,29,30] and that advanced adenomas show specific breath patterns, differing from those expressed in CRC [23]. However, these results must be confirmed, more so in the case of the eNoses, as each sensor is activated by several VOCs belonging to the same chemical class [23,29].

A single study [31] reported that the pattern of exhaled VOCs in CRC patients undergoes modification following cancer resection. The 31 identified VOCs performed very well in discriminating CRC patients before and after surgery and operated patients versus healthy controls. Interestingly, the VOC pattern exhaled by disease-free patients after surgery differed from that of healthy controls, probably to different metabolic pathways induced by surgery- and chemotherapy-induced oxidative stress [31]. The study, carried out in a small series of patients, could not identify changes in the VOC pattern in patients with recurrent or metastatic disease [31].

Conversely, a different research group [28] reported that levels of propanal reverted to normal following surgery, but subsequently increased in the case of CRC recurrence. Using this VOC, the sensitivity and specificity for the identification of recurrence were respectively 71.4% and 90.9% at a threshold of 28 ppbv [28].

To our knowledge, one single study investigated the accuracy of eNoses in detecting extraluminal local recurrences or metastases of CRC [27]. Aeonose™ in this respect showed 88% sensitivity (CI 69–97%) and 75% specificity (CI 57–87%), with an overall accuracy of 81% [27].

Despite an overall good diagnostic accuracy, studies were carried out using different methodologies and identified different VOCs. Thus, the results are not suitable for direct comparison of diagnostic accuracy.

As far as aldehydes are concerned, Altomare and coworkers [33] using GC-MS, identified 15 VOCs significantly differing between CRC and the controls. All of them were nonanal and decanal, but the methodology employed did not allow for the measurement of the more volatile short-chain aldehydes. Propanal, a short-chain compound belonging to this class, has been recently reported to be statistically different between CRC and the controls [28]. Conversely, Amal was unable to identify CRC-specific aldehydes [30].

A specific and reproducible pattern of VOCs to be used in clinical practice are still far from being available.

#### 3.1.2. Fecal VOCs

The efficacy of fecal VOCs in CRC has so far been reported in six studies only, using different approaches (Table 3).

CRC patients were discriminated from controls with 85% sensitivity and 87% specificity (AUC 0.92) with eNose Cyranose 320™ [46]. Interestingly, moderate accuracy was observed when comparing patients with advanced adenomas and controls (sensitivity 62%, specificity 86%), or CRC patients and advanced adenomas (sensitivity 75%, specificity 73%) [46].

Comparable results were reported using SIFT-MS, which differentiated patients affected by CRC and advanced adenoma from healthy controls with an accuracy of 75% (72% sensitivity and 78% specificity) [47]. More recently, the eNose Scent A1 performed even better in a relatively large cohort of patients (86 CRC and 71 controls) (Table 3) [48].

Only two studies used GC-MS and were able to identify specific VOCs [49,50]. Bosch et al. [50] reported that fecal VOCs could discriminate CRC patients from the controls with an AUC of 0.96. Similar accuracy was observed in separating patients with advanced adenomas from the controls, but not between adenoma patients and CRC [50]. Interestingly, the VOC profiles reverted to normal following polypectomy [50].

The other study used gas chromatography coupled to a sulfur chemiluminescence detector and collected VOCs during defecation. However, despite good sensitivity (90%), the overall accuracy (75%) was not superior to that observed by Bosch in stored feces [49].

Finally, some authors [51,52,53] have reported promising results in the detection of CRC-related metabolomic alterations also using different techniques such as nuclear magnetic resonance spectroscopy.

Available data indicate that stool VOCs have acceptable performance in discriminating CRC patients from controls. The studies, however, were carried out in small series using different, non-comparable, techniques. The possibility of identifying advanced adenoma also needs further evaluation.

**Table 3:** Fecal VOCs and colorectal cancers or adenoma detections.

Reference	Aim	Population			Analysis Method	Different VOCs between Groups	Accuracy		
		Intervention Group CRC/Adenoma	CRC Stage (I, II, III, IV)	Control Group			Sens	%Spec	%AUC
Bosch 2020 [50]	Disease detection	14 CRC	n.a.	227	GC-IMS (FlavourSpec^®^)	VOCs Panel	1	1	0.96
	Disease detection	64 AA	n.a.	227			0.96	0.93	0.96
	Disease detection	69 LA	n.a.	227			0.98	0.91	0.96
	Disease detection	127 SA	n.a.	227			0.96	0.93	0.96
	Disease detection	14 CRC	n.a.	260 Adenomas †			n.s.	n.s.	n.s.
Bond 2019 [54]	Disease detection	21 CRC	n.a.	60 ‡	GC-IMS	A, B, C, D, E, F, G, H	0.88	0.85	0.82
Ishibe 2018 [49]	Disease detection	30 CRC	I = 9, II = 12 II = 6, IV = 3	26 HC	GC	I, L	0.90	0.57	0.78
Batty 2015 [47]	Disease detection	31 CRC	n.a.	31 ‡	SIFT-MS	VOCs Panel	0.78	0.72	n.a.
Zonta 2017 [48]	Disease detection	28 CRC and adenomas	n.a.	58	eNose (SCENT A1)	VOCs Panel	0.95	0.95	n.a.
De Meij 2013 [46]	Disease detection	40 CRC	n.a.	57	eNose (Cyranose 320^®^)	VOCs Panel	0.85	0.87	0.92
	Disease detection	60 AA	n.a.	57			0.62	0.86	0.79
	Disease detection	40 CRC	n.a.	60 AA			0.75	0.73	0.82

CRC: colorectal cancer; AA: advance adenoma; LA: large adenoma; SA: small adenoma; n.a.: not available; n.s.: non-significant; HC: healthy control; ‡: cancer screening program; †: AA, LA, and SA; GC: gas chromatography; IMS: ion mobility spectrometry; FAIMS: field asymmetric ion mobility spectrometry; SIFT-MS: selected ion flow tube-mass spectrometry; eNose: electronic nose. A: propan-2-ol; B: hexan-2-0-one; C: ethyl 3-methylbutanoate; D: propan-2-yl butanoate; E: propan-2-yl pentanoate; F: 1,4-xylene; G: propan-2-yl propanoate; H: 5-methyl-2-propan-2-yl-cyclohexan; I: methyl mercaptan; L: hydrogen.

#### 3.1.3. Urinary VOCs

Although the collection of urine samples is easily performed, only six studies investigated urinary VOCs in CRC (Table 4).

FAIMS attained 88% sensitivity and 60% specificity [55], while the “WOLF” eNose showed 78% sensitivity and 79% specificity [56].

However, the diagnostic accuracy of FAIMS-detected urinary VOCs, in a larger cohort including 562 patients, was lower than FIT (63% sensitivity and 63% specificity vs. 80% sensitivity and 93% specificity, respectively) [57]. Unsatisfactory results were observed in high-risk adenomas (specificity of 16%). Combined urinary VOCs and FIT did not provide a significant advantage (80% sensitivity and 89% specificity) [57].

The urinary volatilomes were similar in CRC patients and spouses or first-degree relatives using FAIMS. The VOC profiles became different when the two control groups were grouped, despite far from optimal sensitivity and specificity (69%, 69%, AUC 0.72, *p* < 0.001). This approach minimized the influence of dietary differences in VOC profiles, but differences were not significant in subgroup analysis, possibly due to inadequate statistical power. Despite an interesting approach, no significant advantage over the currently available fecal screening test was observed [58].

In a small mixed cohort [59], GC-MS suggested that high levels of 1,4,5-trimethyl-naphthalene, 2,7-dimethyl-quinoline, and 2-methyl-3-phenyl-2-propenal characterize CRC (Table 4).

The diagnostic accuracy of FAIMS vs. GC-IMS was investigated in a cohort of 163 patients [60], showing an area under the curve (AUC) of 0.98 (95% CI 0.93–1) and 0.82 (95% CI 0.67–0.97), respectively. Neither technique was able to differentiate adenoma patients and the controls [60].

Finally, some authors [61] reported good accuracy in the detection of CRC-related metabolomic alterations also using nuclear magnetic resonance spectroscopy (sensitivity, specificity, and AUC values of 87.5%, 91.3%, and 0.933, respectively) [61].

**Table 4:** Urinary VOCs and colorectal cancers or adenoma detections.

Reference	Aim	Population			Analysis Method	Different VOCs between Groups	Accuracy		
		Intervention Group CRC/Adenoma	CRC Stage (I, II, III, IV)	Control Group			Sens	%Spec	%AUC
McFarlane 2019 [58]	Disease detection	56 CRC	n.a.	82 HC (relatives + spouses)	FAIMS-MS		0.69	0.69	0.71
Widlak 2018 [57]	Disease detection	35 CRC	n.a.	n.a.	FAIMS	VOCs Panel	0.63	0.63	0.67
	Disease detection	27 AA	n.a.	n.a.	FAIMS		0.93	0.16	0.56
	Disease detection	94 A	n.a.	n.a.	FAIMS		0.91	0.15	0.55
Arasaradnam 2014 [55]	Disease detection	83 CRC	65 non-metastatic, 9 metastatic, 9 not-fully staged	50 HC	FAIMS	A, B, C, D, E, F, G, H, I, J, K, L, M, N, O, P, Q, R, S, T, U, V, W	0.88	0.60	n.a.
Mozdiak 2019 [60]	Disease detection	12 CRC	n.a.	12 HC ‡	FAIMS	VOCs Panel	1	0.92	0.98
	Disease detection	12 CRC + 93 A	n.a.	37 HC ‡	FAIMS		0.48	0.89	0.64
	Disease detection	12 CRC + 18 AA	n.a.	37 HC ‡	FAIMS		0.57	0.68	0.62
	Disease detection	12 CRC	n.a.	7 AA ‡	FAIMS		0.83	1	0.92
	Disease detection	10 CRC	n.a.	24 HC ‡	GC-IMS		0.80	0.83	0.82
	Disease detection	10 CRC + 55 adenomas	n.a.	42 HC ‡	GC-IMS		0.71	0.55	0.61
	Disease detection	10 CRC + 13 AA	n.a.	24 HC ‡	GC-IMS		0.48	0.67	0.53
	Disease detection	10 CRC	n.a.	13 AA ‡	GC-IMS		n.a.	n.a.	n.a.
Silva 2011 [59]	Disease detection	12 CRC	n.a.	21 HC	GC-MS	a, b, c, d, e, f, g, h, i, j	n.a.	n.a.	n.a.
Westenbrink 2015 [56]	Disease detection	39 CRC	n.a.	35 IBS	e-Nose (Warwick OLFaction ^®^)	VOCs Panel	0.78	0.79	n.a.

CRC: colorectal cancer; AA: advance adenoma; A: adenoma; n.a.: not available; HC: healthy control; ‡: cancer screening program; GC: gas chromatography; IMS: ion mobility spectrometry; FAIMS: field asymmetric ion mobility spectrometry; eNose: electronic nose. A: acetaldehyde; B: ethylene oxide; C: oxalic acid; E: dimethyl diazene; F: cyclobutyl amine; G: oxepane; H: acetone; I: 2-pentanone; J: 3-methyl-2-butanone; K: 2,3-butanedione; L: 4-heptanone; M: 3-heptanone; 2,4-dimethyl-3-pentanone, acetyloxime-pyridine carboxaldehyde, hydrocinnamoyl-bezene-ethanamine, styrene, N: dimethyl-thiourea; O: allyl isothiocyanate; P: isothiocyanato-cyclopropane; Q: 2-cyano-acetamide; R: methoxy-phenyl-oxime; S: ethylbenzoic acid (pentyl ester); T: carbamic acid (methyl ester); U hexen-1-ol; V: 4-methyl-1-hexene; W: hexanal; a: p-cymene; b: anisole; c: g-terpinene; d: bornylene; e: dimethyl disulfide; f: 4-methylphenol; g: 1,2-dihydro-1,1,6-trimethylnaphthalene; i: 1,4,5-trimethylnaphthalene; j: 2,7-dimethylquinoline.

### 3.2. Inflammatory Bowel Diseases

#### 3.2.1. Breath Exhaled VOCs

In IBD, endoscopy represents the gold standard for the diagnosis and the assessment of disease activity as well as for the detection of the related dysplasia and CRC. Being expensive and invasive, the procedure is not well accepted by many patients. Nonetheless, periodic monitoring is essential to assess the progression of mucosal inflammation [62], optimize treatment, and prevent complications. To reduce the need for endoscopy, several non-invasive biomarkers are routinely used in clinical practice, but all show limitations [5,6]. Thus, alternative strategies such as VOC analysis are needed (Table 5).

The proposed mechanism of VOC production in IBD is largely dependent on oxidative stress causing lipid peroxidation. Several studies suggested the potential role of single breath exhaled VOCs such as pentane and hexane as markers of IBD [36,41,42,43]. The potential role of VOC patterns including hydrogen sulfide, acetic acid, propanoic acid, and butanoic acid has also been suggested, as higher than normal concentrations are present in IBD patients [36]. The correlation of these putative markers with clinical indexes (Harvey–Bradshaw Index for CD and Simplified Clinical Colitis Activity Index for UC) is unfortunately weak [36].

Acceptable accuracy in distinguish IBD from the controls (74% sensitivity, 75% specificity, AUC 0.82), and UC from CD (67% sensitivity, 67% specificity, AUC 0.70) has also been reported [38]. The results are in line with those previously reported by Hicks et al. [39], who recorded different VOCs (Table 1) in a small cohort of patients. In this study, despite being able to distinguish both CD and UC from healthy controls (AUC 0.86 and 0.74, respectively), and CD from UC (AUC 0.83), VOC analysis did not differ significantly in active versus inactive disease [39].

Specific VOC profiles, analyzed by GC-MS, were used in a larger cohort of adult CD patients to distinguish healthy controls from active and inactive CD [40]. Two sets of six discriminatory compounds (Table 1) were identified, showing sensitivity 96%, specificity 99%, AUC 0.99, and sensitivity 96%, specificity 97%, and AUC 0.98, respectively. A panel of 10 VOCs proved to be effective in separating active from inactive disease (sensitivity 81%, specificity 80%, AUC 0.88) [40]. The reliability of results was far from optimal, as disease activity was not assessed by endoscopy, but was defined based on fecal calprotectin, c-reactive protein, and the Harvey–Bradshaw Index.

Similar results were reported by the same group in UC, using a set of 11 VOCs to discriminate clinically active from inactive disease (92% sensitivity and 77% specificity) [35]. Again, however, endoscopy was not performed, only fecal calprotectin [35].

The accuracy of the VOC pattern in the diagnosis of IBD was also assessed using eNoses [15].

A small study compared the performance of an eNose and gas chromatography–ion mobility, reporting that both separated IBD from the controls (eNose: AUC 0.81, sensitivity 67%, specificity 89%; GC-IMS: AUC 0.93, sensitivity 87%, specificity 89%) as well as CD from UC (eNose: AUC 0.88, sensitivity 71%, specificity 88%; GC-IMS: AUC 0.71, sensitivity 86%, specificity 62%) [15]. The measurement of fecal calprotectin did not improve the diagnostic accuracy of breath analysis [15].

No link was found between VOC levels and IBD complications [37]. Similarly, VOCs proved inadequate for identifying disease location and activity and did not correlate with laboratory parameters and therapy. The only exception was represented by the VOC patterns of patients with ileal-pouch-anal-anastomosis, which differed from that from all other groups but, again, proved unable to detect pouch inflammation [37].

Similar results have been reported in pediatric IBD. Three discriminatory VOCs were effective in separating IBD patients from healthy controls but did not correlate with the degree of inflammation [45].

These results have been confirmed by a recent Italian study, reporting good sensitivity, but low specificity in distinguishing IBD from the controls, and CD from UC [44] (Table 5). Again, no relevant differences were observed with disease activity.

In conclusion, despite differences in the sampling methods and measurement techniques, breath analysis seems to be overall effective in separating IBD patients from the normal controls. It may thus help to select patients with the suspected disease, who should undergo endoscopy. The most relevant clinical application of VOC analysis is, however, the assessment of disease activity, to avoid an unnecessary colonoscopy. In this respect, the available evidence is conflicting (Table 5) and largely based on studies flawed by the lack of endoscopy and histology.

Chronic long-standing IBD increases the risk of CRC [63]. The possibility of identifying high-grade dysplasia by non-invasive techniques by the identification of specific VOCs-patterns would be of prime clinical importance. No data, however, are presently available in the literature.

**Table 5:** Breath test VOCs and IBD diagnosis and activity evaluation.

Reference	Aim	Population		Analysis Method		Different VOCs between Groups	Accuracy		
		Intervention Group (Samples Collected) [CD; UC]	Control Group (Samples Collected)		Sample Collection Method		Sens%	Spec%	AUC
Arasaradnam 2016 [55]	Disease diagnosis	54 [25 CD; 29 UC]	22 HC	FAIMS	Tedlar^®^ bags	n.a.	0.74	0.75	0.82
	Differential diagnosis	25 CD	29 UC			n.a.	0.67	0.67	0.70
Tiele 2019 [15]	Disease diagnosis	30 IBD [14 CD; 16 UC]	9 HC	GC-IMS	Direct measurement	A, B	0.87	0.89	0.93
				eNose (Warwick OLFaction)	Bio-VOC sampling device	.	0.67	0.89	0.81
	Differential diagnosis	14 CD	16 UC	GC-IMS		.	0.86	0.62	0.71
				eNose (Warwick OLFaction)		.	0.71	0.88	0.88
Smolinska 2017 [35]	Disease activity	UC remission (70)	UC active (62)	GC-tof-MS	Tedlar bags	C, D, E, F, G	0.92	0.77	0.94
Bodelier 2015 [40]	Disease diagnosis	140 active CD (*725 †)*	110 HC (*110*)	GC-tof-MS (and PCA)	Tedlar bags	VOCs Panel ¶	0.96	0.99	0.99
	Disease diagnosis	135 inactive CD (725 †)	110 HC (*110*)			VOCs Panel ¶	0.96	0.97	0.98
	Disease activity	140 active CD	135 remission CD			VOCs Panel ¶	0.81	0.80	0.88
Pelli 1999 [42]	Disease diagnosis	10 CD	10 HC	GC	Tedlar bags	H	n.a.	n.a.	n.a.
	Disease diagnosis	10 UC	10 HC			H	n.a.	n.a.	n.a.
Sedghi 1994 [43]	Disease diagnosis	17 UC (56)	14 HC	GC	Plastic syringes	Z	n.a.	n.a.	n.a.
Dryahina 2017 [36]	Disease diagnosis	187 IBD [136 CD; 51 UC]	14 HC	SIFT-MS	Nalophan bags	F, H, I, L, M, N, O, P, Q	n.a.	n.a.	n.a.
Rieder 2016 [37]	Disease diagnosis	36 IBD [24 CD; 11 UC]	53 HC	SIFT-MS	Mylar bag	I, L, M, R, S, T, U	n.a.	n.a.	n.a.
	Disease diagnosis	36 IBD [24 CD; 11 UC]	6 OGDs			I, M, V	n.a.	n.a.	n.a.
	Disease activity	n.a.	n.a.			VOCs Panel ¶ **	n.a.	n.a.	n.a.
	Differential diagnosis	24 CD	11 UC			VOCs Panel ¶ **	n.a.	n.a.	n.a.
Hicks 2015 [39]	Disease diagnosis	18 CD	18 HC	SIFT-MS (and PCA and OSC-PLS-DA)	Nalophan bags	N, T, W, X	0.94	0.94	0.86
	Disease diagnosis	20 UC	18 HC			V	0.90	0.94	0.74
	Differential diagnosis	18 CD	20 UC			N, W, Y	0.89	0.90	0.82
Dryahina 2013 [41]	Disease diagnosis	20 CD	140 HC	SIFT-MS	Direct measurement	H	n.a.	n.a.	n.a.
	Disease diagnosis	28 UC	140 HC			H	n.a.	n.a.	n.a.
Monasta 2017 [44] (pediatric patients)	Disease diagnosis	67 IBD (124) [34 CD; 33 UC]	167 (334) [102 HC; 65 GIC]	IMR-MS	Bio-VOC sam-pling device	VOCs Panel ¶	0.95	0.69	0.92
	Differential diagnosis	34 CD	33 UC			VOCs Panel ¶	0.94	0.71	0.88
Patel 2014 [45] (pediatric patients)	Disease diagnosis	62 IBD [51 CD; 11 UC]	55 HC	SIFT-MS	Mylar bags	Aa, Ab, Ac	n.a.	n.a.	0.96
	Differential diagnosis	51 CD	11 UC			VOCs Panel ¶ **	n.a.	n.a.	n.a.

UC: ulcerative colitis; CD: Crohn’s disease; IBD-U: undetermined inflammatory bowel disease; HC: healthy control; GIC: gastrointestinal control; OGDs: other gastrointestinal diseases; †: both active and inactive; ¶: shown in Table 1; **: *p* > 0.05, GC: gas chromatography; IMS: ion mobility spectrometry; SIFT-MS: Selected ion flow tube-mass spectrometry GC-tof-MS: gas-time of flight-mass spectrometry; IMR-MS: ion-molecule reaction-mass spectrometry; FAIMS: field asymmetric ion mobility spectroscopy; OSC-PLS-DA: orthogonal signal correction—partial least squares discriminant analysis; PCA: principal components analysis, A: 2-methyl-,propyl ester; B: 3-methyl-1-butyl ester; C: 2,4-dimethylpentane; D: methylcyclopentene; E: octane; F: acetic acid; G: m-cymene; H: pentane; I: 2-propanol; L: isoprene; M: ethanol; N: hydrogen sulfide; O: acetone; P: propanoic acid; Q: butanoic acid; R: acetonitrile; S: carbon disulfide; T: dimethyl sulfide; U: triethyl amine; V: ammonia; W: butanal; X: nonal; Y: hydrogen cyanide; Z: ethane; Aa: 1-octene; Ab: 1-decene; Ac: (E)-2-nonene.

#### 3.2.2. Fecal VOCs

The few available data suggest that fecal VOCs differ in IBD patients and controls [64,65,66] (Table 6). This holds in differentiating IBS from IBD in both pediatric patients (AUC of 0.94) [64] and in adults versus IBS-D, using GC-MS [67]. The sensitivity was 94% and 96%, and specificity 82% and 80%, respectively, for CD and UC [67].

The diagnostic accuracy was slightly varied with the different techniques used or differing study groups (Table 6) [68].

Only one study using eNose technology was aimed at differentiating CD from UC [69]. The VOC profile in CD patients differed from that in UC, both during active disease (AUC 0.96; sensitivity 97%; specificity 92%) and clinical remission (AUC 0.81; sensitivity 88%; specificity 72%) [69].

Again, studies were carried out in small cohorts, more so when focusing on disease activity. To our knowledge, no data are available on IBD complications, especially for IBD-related CRC.

**Table 6:** Fecal VOCs and IBD diagnosis and activity evaluation.

Reference	Aim	Population		Analysis Method	Different VOCs between Groups	Accuracy		
		Intervention Group (Samples Collected) [CD; UC]	Control Group (Samples Collected)			Sens%	Spec%	AUC
Bosch 2020 [50]	Disease diagnosis	276 IBD (495) †	227 HC (227)	GC-IMS (FlavourSpec^®^)	VOCs Panel	0.97	0.92	0.96
	Disease diagnosis	164 CD (292) †	227 HC (227)			0.96	0.97	0.96
	Disease diagnosis	112 UC (197) †	227 HC (227)			0.91	0.88	0.95
	Differential diagnosis	164 CD (187) †	112 UC (147)			0.17	0.96	0.55
	Disease activity	active CD (107)	inactive CD (84)			0.76	0.43	0.52
	Disease activity	active UC (80)	inactive UC (63)			0.67	0.57	0.63
Shepherd 2014 [70]	Disease diagnosis	101 IBD	46 HC	GC	VOCs Panel	0.78	0.80	n.a.
Ahmed 2013 [67]	Disease diagnosis	110 IBD	30 IBS	GC-MS	A, B, C, D, E, F, G, H, I, J, K	0.96	0.80	n.a.
	Disease diagnosis	62 CD	30 IBS			1	0.80	n.a.
	Disease diagnosis	48 UC	30 IBS			0.94	0.87	n.a.
El Manouni El Hassani 2019 [68] (Pediatric patients)	Differential diagnosis	17 IBD [15 CD; 2 UC]	25 HC	FAIMS	VOCs Panel	0.94	0.96	0.99
Bosch 2018 [65] (paediatric patients)	Disease diagnosis	30 IBD [15 CD; 15 UC]	15 IBS-FAP/NOS	FAIMS	VOCs Panel	1	0.87	0.94
	Disease diagnosis	30 IBD [15 CD; 15 UC]	30 HC			0.93	0.97	0.96
	Disease diagnosis	15 CD	30 HC			0.93	0.93	0.95
	Disease diagnosis	15 UC	30 HC			0.93	0.97	0.98
	Differential diagnosis	15 CD	15 UC			0.60	0.80	0.67
Van Gaal 2017 [66] (Pediatric patients)	Disease diagnosis	36 IBD [23 CD; 13 UC]	24 HC	FAIMS	VOCs Panel	0.79	0.78	0.76
	Disease diagnosis	23 CD	24 HC			0.83	0.83	0.90
	Disease diagnosis	13 UC	24 HC			0.77	0.75	0.74
	Differential diagnosis	23 CD	13 UC			n.s.	n.s.	n.s.
Bosch 2018 [64] (paediatric patients)	Disease diagnosis	10 IBD (*10*) [5 CD; 5 UC]	10 OGDs (*10*)	GC-IMS (FlavourSpec^®^)	VOCs Panel	0.70	0.90	0.73
De Meij 2014 [69] (Paediatric patients)	Disease diagnosis	active UC (26)	28 HC	eNose (Cyranose 320^®^)	VOCs Panel	1	1	1
	Disease diagnosis	Inactive UC (17)	28 HC			0.94	0.94	0.94
	Disease diagnosis	Active CD (6)	28 HC			0.87	0.67	0.85
	Disease diagnosis	Inactive CD (20)	28 HC			0.94	0.94	0.94
	Differential diagnosis	Active CD (6)	Active UC (12)			0.97	0.92	0.96
	Differential diagnosis	Inactive CD (20)	Inactive UC (17)			0.86	0.72	0.81

UC: ulcerative colitis; CD: Crohn’s disease; HC: healthy control; OGDs: other gastrointestinal diseases; IBS-FAP/NOS: irritable bowel syndrome-functional abdominal pain/not-otherwise specified; †: both active and inactive; GC: gas chromatography; IMS: ion mobility spectrometry; FAIMS: field asymmetric ion mobility spectrometry; eNose: electronic nose. A: 2-methylpropanal; B: undecane; C: heptanal; D: methylbutanal; E: isopropyl alcohol; F: 2-methyl,1-propanol; G: cyclohexene; H: methoxy-phenyl-oxime; I: butanoic acid; J: 3-methyl-S-methyl ester; K: 2-methyl-, ethyl ester.

#### 3.2.3. Urinary VOCs

Data on urinary VOCs in IBD are scarce (Table 7). VOC differences in urine samples of IBD patients and controls were first reported in 2009 using an eNose [71]. The samples of a small cohort of 48 IBD patients were subsequently analyzed with both eNose and FAIMS by the same authors. Both technologies were able to separate IBD patients from controls with 88% and 75% accuracy, respectively (*p* < 0.001), and active from the inactive disease [72].

GC-IMS detected significantly different VOC profiles in 10 IBD patients vs. 10 controls (*p* = 0.028, AUC = 0.78) [68]. This small study reported better performance of urinary-compared to fecal VOCs, as far as sensitivity was concerned (80% vs. 70%), but not for specificity (70% vs. 90%).

Urinary metabolomic profiles were studied by a combination of direct infusion liquid chromatography/tandem mass spectrometry and GC-MS assays [16]. UC patients in remission differed from IBS patients with AUC 0.99, specificity 99.9%, and sensitivity 99.8% (*p* < 0.001).

Additionally, in this field, some reports have suggested that NMR might prove useful in identifying metabolomic alterations [73,74].

## 4. Discussion

VOCs are the volatile fraction of metabolome, resulting from the ongoing metabolic processes of gut bacteria. These compounds can be detected in breath, urine, and feces [75] (Figure 1). Specific VOC patterns reflect health status and are supposed to undergo disease-specific changes [76].

Volatilome is influenced by exogenous and endogenous factors such as environmental diet and pollution, which significantly contribute to the pattern of production of VOCs [77]. Moreover, VOC profiles in CRC and IBD result from inflammation-induced dysbiosis [12,78] as well as medications directly affecting the gut microbiome such as antibiotics and proton pump inhibitors [79].

Bacterial enzymatic activities are also modulated by the microenvironmental pH. The intracolonic pH has been demonstrated to be higher than normal in CRC and lower in IBD compared to a healthy subject, thus affecting enzymatic activities and composition of bacterial metabolites [80,81,82]. The bowel preparation before endoscopy is also relevant. Thus, the relative importance of individual factors and their complex interactions need further investigation [23,83].

Nonetheless, VOC analysis represents an attractive, non-invasive method to evaluate patient health status and possibly diagnose pathological conditions. In intestinal diseases, endoscopy still represents the mainstay of diagnostic workup, but it is invasive and expansive. If feasible, this novel approach would represent an attractive alternative to endoscopy. Aside from avoiding unnecessary procedures, VOC analysis may result in a reduction in health care costs. Indeed, this novel diagnostic approach is relatively unexpansive, as fecal VOC profiling costs approximately €11 [35], 30% less than fecal occult blood testing [72]. Urinary VOC evaluation cost is slightly more expensive, twice as much as a fecal immunochemical test (28 vs. 18 £/test) [55,57].

However, VOC analysis is not standardized and is still far from clinical application. Most data refer to breath sampling, which is easy to perform and easily accepted by patients, while the analysis of fecal and urine samples, although attractive, does not represent valuable alternative strategies. Moreover, analysis of fecal samples may prove a less effective choice. Given the suboptimal compliance to the standard fecal occult blood test as a screening procedure, the same can be anticipated for fecal VOC analysis. Urine samples may be more easily accepted by patients.

Technical hurdles such as the lack of standardization in sample collection, different VOC profiles, and measurement techniques are shared by VOC analysis, irrespective of the sampling of alveolar air, stool, or urine. Urine and feces, moreover, need to be processed, stored within few hours from collection, and then be defrosted and warmed-up to obtain the headspace for analysis.

Storage significantly affects the more volatile compounds, but not necessarily the results [36,84]. The diagnostic accuracy of fecal and urinary VOCs was not significantly influenced by storage duration (20 months for fecal and 12 months for urinary VOCs) in some studies [65,85]. Other authors have reported that different sampling conditions and sample characteristics (sample mass, temperature, water content, time at room temperature before storage) may instead affect the outcome [86]. Optimal stool sampling and storage conditions have not yet been defined, but freezing 500 mg fecal samples diluted with 10 mL of tap water, thawing 10 min before analysis, and a single thaw freeze cycle showed the best accuracy for disease detection in IBD [65]. It is unclear whether the same applies to CRC.

**Figure 3:**
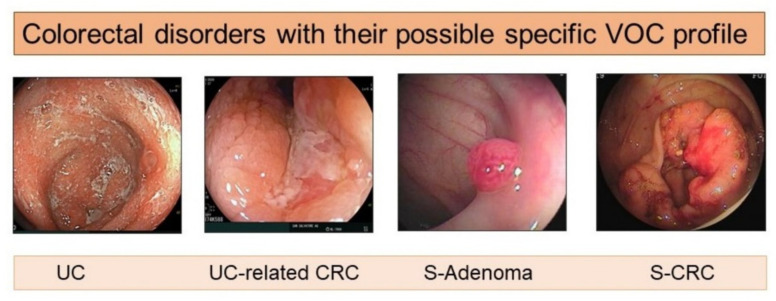
Increased evidence suggests that patients with inflammatory bowel disease (IBD), both ulcerative colitis (UC) and Crohn’s Disease (CD), IBD-related CRC, sporadic adenomas, or sporadic CRC may have their own specific VOC profile that could be used in their diagnosis and monitoring. It is not known whether VOCs have a pathogenetic role in colorectal carcinogenesis. S-adenoma = sporadic adenoma; S-CRC = sporadic colorectal cancer; VOCs = volatile organic compounds.

VOCs include a large number of different chemical compounds, largely overlapping in health and disease. Thus, statistics using principal component analysis (PCA) and probabilistic neural network (PNN) analysis is required for reducing a large set of variables to small ones. This, in turn, further contributes to the variability of the results reported in different studies. Thus, the results and interpretation of data often differ in different settings despite the use of the same instrumentation and comparable series of patients, more so using different procedures based on GC-MS versus eNoses. Consequently, distinct VOC profiles have been identified in the same disease, thus far preventing the use of VOC analysis in clinical practice. Nonetheless, some stimulating information is growing in specific conditions such as IBD, IBD-related CRC, sporadic colorectal adenomas, and sporadic CRC (Figure 3).

In cancer, most data are derived from small-powered pilot studies, inadequate to validate the test as a mass screening tool. In IBD, patients are mainly compared with healthy volunteers.

Despite acceptable accuracy in separating IBD patients from the controls, the methodology is still far from useful clinical use in IBD. Specific VOC patterns pinpointing active inflammation, fibrostenosing, or fistulizing phenotype in CD and cancer risk in UC are lacking.

Despite all the above-mentioned shortcomings, VOC analysis is promising, considering that recent advances render VOC analysis progressively more effective for early diagnosis of colorectal cancer. Being non-invasive, time- and cost-sparing, and safe, this approach may help improve compliance in screening programs and possibly replace fecal occult blood testing.

Combination of differing VOC panels, and improving the performance of diagnostic algorithms will enhance accuracy. Refining analytical methods will also allow more accurate detection and quantification of specific metabolites, rendering VOC analysis a suitable tool for the diagnosis and management of several diseases, especially CRC and IBD.

## 5. Conclusions

Despite all the above-mentioned shortcomings, VOC analysis could be reasonably effective in separating IBD or CRC patients from the controls (Figure 3).

Future studies with more robust methodology, carried out in suitably large cohorts of patients are needed to validate the currently available evidence. Focus studies on the interrelation between bacterial metabolism, disease, and specific VOC pattern, and the mechanism leading to their production are needed to explore the full potential of this novel tool.

Besides identifying specific VOCs pattern in adenoma, sporadic CRC, and IBD in the presence or absence of high-grade dysplasia, it shall be crucial to establish the relationship between VOCs and the underlying disease. It is still unclear whether metabolic processes leading to differing VOC panels are a consequence of disease or represent a contributing factor to its development. Furthermore, no prospective studies have assessed the risk of CRC development by using VOCs in samples collected before the onset of disease, both in the general population and in patients with IBD.

## Figures and Tables

**Table 1:** All breath test VOCs analyzed in each specific study.

	Colorectal Carcinoma (CRC)
References	Identified VOCs
Altomare 2020 [26]	Tetradecane; ethylbenzene; methylbenzene; 5,9-undecadien-2-one + 6,10-dimethyl (E); tridecane; benzaldehyde; dodecane; benzoic acid; 1,3-bis(1-methylethenyl) benzene; decanal; 2-ethyl-hexanol; ethenone + 1[4-(1-methylethenyl) phenyl]; acetic acid; butyl hydroxy toluene; unknown VOC.
Steenhuis 2020 [27]	n.a.
Van Keulen 2020 [23]	n.a.
Markar 2019 [28]	Propanal; ammonia; ethanol; acrolein; propanol; butanol; carbon disulfide.
Altomare 2016 [29]	Methane.
Amal 2016 [30]	Acetone; ethyl acetate; 4-methyl-octane; ethanol.
Altomare 2015 [31]	1,2-pentadiene; 2-methylbutane; 3-ethylpentane; methylcyclo-pentane; cyclohexane; nonanal; methylcyclohexane; 4-methyl-2-pentanone; 1,4-dimethylbenzene; 1,3-dimethylbenzene; decanal.
Wang 2014 [32]	Cyclohexanone; 2,2-dimethyldecane; 4-ethyl-1-octyn-3-ol; ethylaniline; cyclooctylmethanol; trans-2-dodecen-1-ol; 3-hydroxy-2,4,4-trimethylpentyl 2-methylpropanoate; dodecane.
Altomare 2013 [33]	4-methyl-octane; 1,2-pentadiene; 2-methylbutane; cyclohexane; nonanal; methylcyclohexane; 4-methyl-2-pentanone; 1,4-dimethylbenzene; 1,3-dimethylbenzene; 2-methylpentane; 3-methylpentane; 4-methylundecane; trimethyldecane; decanal.
Peng 2010 [34]	1,3-dimethylbenzene; 1,10-(1-butenylidiene) bis-benzene; 1-iodonane; [(1,1-dimethylethyl) thio] acetic acid; 4-(4-propylciclohexyl)-4′-cyano [1,10-biphenyl]-4-yl ester benzoic acid; 2-amino-5-isopropyl-8-methyl-1-azulenecarbonitrile.
	**Inflammatory Bowel Disease (IBD)**
	**Identified VOCs**
Tiele 2019 [15]	2-methyl-, propyl ester; 3-methyl-1-butyl ester
Smolinska 2017 [35]	Unknown VOCs; cumene; 2,4-dimethylpentane; methylcyclopentene; C_14_H_30_ branched; C_15_H_30_ (pentadecene); 3-methyl-1-butanlo, octane, acetic acid, α-pinene; m-cymene.
Dryahina 2017 [36]	Pentane; isoprene; ethanol; propanol; hydrogen sulfide; acetone; acetic acid; propanoic acid; butanoic acid.
Rieder 2016 [37]	2-propanol; acetaldehyde; acetone; acetonitrile; acrylonitrile; benzene; carbon disulfide; dimethyl sulfide; ethanol; isoprene; pentane; 1-decene; 1-heptene; 1-nonene; 1-octene; 3-methylhexane; (e)-2-nonene; ammonia; ethane; hydrogen sulfide; triethyl amine; trimethyl amine.
Arasaradnam 2016 [38]	n.a.
Hicks 2015 [39]	Acetic acid; pentanoic acid; hexanoic acid; propanal; butanal; pentanal; hexanal; heptanal; octanal; nonanal; decanal; methanonal; propanol; butanol; pentanol; phenol; methyl phenol; Ethyl phenol; acetone; dimethyl sulfide; dimethyl disulfide; hydrogen sulfide; carbon disulfide; ammonia; hydrogen cyanide; isoprene
Bodelier 2015 [40]	Isoprene; acetone; 2,2,4-trimethylpentane; heptadecane; saturated C12; 1-butoxy-2-propanol; unknown VOC (healthy control vs. active Crohn’s disease)—isoprene; acetone; heptadecane; undecanal; two unknown VOCs (healthy control vs. remission Crohn’s disease)—decanal; 1-hydroxy-2-propanone; hexadecanal; 2,2,4-trimethylhexane; 2,2,4,4-tetramethyloctane; acetic acid methyl ester; four unknown VOCs (active vs. remission Crohn’s disease)
Dryahina 2013 [41]	Pentane
Pelli 1999 [42]	Ethane; Propane; Butane; Pentane; Isoprene
Sedghi 1994 [43]	Ethane; Pentane
Monasta 2017 [44] (pediatric patients)	Methane *; ammonia; propene *; acetonitrile ł; nitrous oxide ł; nitrous acid *; acetic acid *; methyl ethyl ketone; methanimineł; cyclopentane ł; carbon disulfide *; methyl nitrate ł; pyridine; methylpyrrole *; ethyl cyanoformate *; dimethyylpyridine; trimethylpentane ł; ammonia; ethylene; acetaldehyde; acetone; isoprene; toluene; n-heptane.
Patel 2014 [45] (Pediatric patients)	2-propanol; acetaldhyde; acetone; acrylonitrile; benzene; carbon disulfide; dimethyl sulfide; ethanol; isoprene; pentane; 1-decene; 1-heptene; 1-nonene; 1-octene; 3-methylhexane; (E)-2-nonene; ammonia; ethane; hydrogen sulfide; triethyl amine; trimethyl amine.

* IBD vs. control only; ł: Crohn’s disease vs. ulcerative colitis only; n.a.: not available.

**Table 2:** Breath test VOCs and colorectal cancers or adenoma detections.

Reference	Aim	Population			Analysis Method	Sample Collection Method	Different VOCs between Groups	Accuracy		
		Intervention Group CRC/Adenoma	CRC Stage (I, II, III, IV)	Control Group				Sens	%Spec	%AUC
Altomare 2020 [26]	Disease detection	83	I–II = 38 III–IV = 42 n.a. = 3	90	GC-MS	ReCIVA^®^ sorbent tubes	VOCs Panel ¶	0.90	0.93	0.98
		38 (early cancer)	I–II = 38	90			VOCs Panel ¶	0.86	0.94	0.98
Amal 2016 [30]	Disease detection	65 CRC	CIS = 1 I = 21 II = 22 III = 18 IV = 2 n.a. = 1	122	GC-MS	Tedlar bags	A, B, C, D (lower in CRC).	0.85	0.94	n.a.
Altomare 2015 [31]	POR detection	32 in FU	I–II = 20 III–IV = 12	55	GC-MS	Tedlar bags	VOCs Panel ¶	1	0.96	0.96
				32 CRC in FUAS			VOCs Panel ¶	1	0.95	1
Wang 2014 [32]	Disease detection	20 CRC	I–II = 12 III = 8	20	GC-MS	Gas-tight syringe and glass vials	VOCs Panel ¶ E (lower in CRC).	n.a.	n.a.	n.a.
Altomare 2013 [33]	Disease detection	37 CRC	I–II = 19 III–IV = 18	41	GC-MS	Tedlar bags	VOCs Panel ¶	0.86	0.83	0.85
Peng 2010 [34]	Disease detection	26 CRC	PM = 2 I–II = 10 III–IV = 14	22	GC-MS	Mylar polyvinyl fluoride bags	VOCs Panel ¶	n.a.	n.a.	n.a.
Markar 2019 [28]	Disease detection	50 CRC	I = 9, II = 15 III = 18, IV = 1	50 *	SIFT-MS	Direct measurement	VOCs Panel ¶	0.90	0.66	0.83
				50 HC				0.96	0.76	0.90
		25 CRC	n.a.	54			F	0.83	0.84	0.79
	POR detection	21 POR		19 CRC (no POR)			F	0.71	0.90	0.81
Steenhuis 2020 [27]	Disease detection	62 in FU	I = 13, II = 12, III = 19, IV = 18	n.a.	eNose Aeonose^®^	Direct measurement	n.a.	0.88	0.75	0.86
Van Keulen 2020 [23]	Disease detection	62 CRC	I = 22, II = 22 III = 23, IV = 3	104	eNose Aeonose^®^	Direct measurement	n.a.	0.95	0.64	0.84
		174 (CRC + AA)		104				0.83	0.54	0.72
Altomare 2016 [29]	Disease detection	15 CRC, 15 polyps	I = 1, II = 12 III = 2	15	eNose PEN3	Tedlar Bags	G	0.93	0.10	n.a.

CRC: colorectal cancer; HC: healthy control; CIS: carcinoma in situ; AA: advance adenoma; NAA: non-advance adenoma; * IBD, diverticular disease and polyps; n.a.: not available; POR: post-operative recurrence; FU: follow-up; FUAS: follow-up after surgery; PM: premalignant; ¶: VOCs panel for each study is shown in Table 1. GC: gas chromatography; GS-MS: gas chromatography-mass spectrometry; IMS: ion mobility spectrometry; SIFT-MS: selected ion flow tube—mass spectrometry. A: acetone; B: ethylacetate; C: 4-methyl-octane; D: ethanol; E: 6-t-butyl-2,2,9,9-tetramethyl-3,5-decadien-7-yne F: propanal; G: methane.

**Table 7:** Urinary VOCs and IBD diagnosis and activity evaluation.

Reference	Aim	Population		Analysis Method	Different VOCs between Groups	Accuracy		
		Intervention Group [CD; UC]	Control Group			Sens%	Spec%	AUC
Keshteli 2019 [16] *	Disease diagnosis	53 IBD [0;53]	39 IBS	GC-MS	A, B, C, D, E, F, G, H, I, J, K, L, M, N	0.99	0.99	0.99
El Manouni El Hassani 2019 [68] (Paediatric patients)	Differential diagnosis	10 IBD [5 CD; 5 UC]	10 HC	GC-IMS	VOCs Panel	0.80	0.70	0.78
Arasaradnam 2013 [72]	Disease diagnosis	48 IBD [24 CD; 24 UC]	14 HC	FAIMS	VOCs Panel	n.a.	n.a.	n.a.
	Disease diagnosis	48 IBD [24 CD; 24 UC]	14 HC	e-nose (Fox 4000^®^)	VOCs Panel	n.a	n.a.	n.a.

UC: ulcerative colitis; CD: Crohn’s disease; HC: healthy control; GC: gas chromatography; IMS: ion mobility spectrometry; FAIMS: field asymmetric ion mobility spectrometry; eNose: electronic nose. A: lactic acid; B: proline; C: oxoglutaric acid; D: glutamic acid; E: ethylmalonic acid; F: 3-hydroxyisovaleric acid; G: citrulline; H: hydroxyphenylacetic acid; I: adipic acid; J: histidine; K: lysine; L: glutamine; M: phenylalanine; N: Sumiki’s acid. *: Some of the cited compounds are not particularly volatile.

## Data Availability

No supporting data is available.
